# Thrombin Generation and D-Dimer for Prediction of Disease Progression and Mortality in Patients with Metastatic Gastrointestinal Cancer

**DOI:** 10.3390/cancers14184347

**Published:** 2022-09-06

**Authors:** Cinzia Giaccherini, Cristina Verzeroli, Laura Russo, Sara Gamba, Carmen Julia Tartari, Silvia Bolognini, Francesca Schieppati, Chiara Ticozzi, Roberta Sarmiento, Luigi Celio, Giovanna Masci, Carlo Tondini, Fausto Petrelli, Francesco Giuliani, Andrea D’Alessio, Filippo De Braud, Armando Santoro, Roberto Labianca, Giampietro Gasparini, Marina Marchetti, Anna Falanga

**Affiliations:** 1 Immunohematology and Transfusion Medicine, Hospital Papa Giovanni XXIII, 24127 Bergamo, Italy; 2 Oncology Unit, Hospital San Filippo Neri, 00135 Rome, Italy; 3 Oncology Unit, IRCCS National Cancer Institute, 20133 Milan, Italy; 4 Oncology Unit, IRCCS Humanitas Institute, 20089 Rozzano, Italy; 5 Oncology Unit, Hospital Papa Giovanni XXIII, 24127 Bergamo, Italy; 6 Oncology Unit, Hospital Treviglio-Caravaggio, 24047 Treviglio, Italy; 7 Oncology Unit, IRCCS Cancer Institute Giovanni Paolo II, 70124 Bari, Italy; 8 Oncology Unit, San Paolo ASL Bari, 70132 Bari, Italy; 9 Medical Oncology and Internal Medicine, Policlinico San Marco, Gruppo San Donato, 24040 Zingonia-Bergamo, Italy; 10 Medical Oncologist, 24100 Bergamo, Italy; 11 School of Medicine, University of Milano Bicocca, 20126 Milan, Italy

**Keywords:** gastrointestinal cancer, hypercoagulability, prognosis, survival, D-dimer, thrombin generation

## Abstract

**Simple Summary:**

Assessing the prognosis of a patient with cancer is of the greatest clinical interest and can provide useful information on the type and intensity of the anticancer treatment to be administered. The emerging predictive role of coagulation biomarkers in cancer prognosis needs to be confirmed by prospective cohort studies. In this study, we evaluated whether prechemotherapy levels of thrombotic biomarkers may predict for early disease progression and overall survival in a large prospective cohort of patients with metastatic gastrointestinal cancer specifically enrolled for the intended aims. We found that pretreatment thrombin generation and D-dimer appear to be promising candidate biomarkers for both outcomes.

**Abstract:**

Background: the tight and reciprocal interaction between cancer and hemostasis has stimulated investigations on the possible role of hemostatic biomarkers in predicting specific cancer outcomes, such as disease progression (DP) and overall survival (OS). In a prospective cohort of newly diagnosed metastatic gastrointestinal (GI) cancer patients from the HYPERCAN study, we aimed to assess whether the hemostatic biomarker levels measured before starting any anticancer therapy may specifically predict for 6-months DP (6m-DP) and for 1-year OS (1y OS). Methods: plasma samples were collected and tested for thrombin generation (TG) as global hemostatic assay, and for D-dimer, fibrinogen, and prothrombin fragment 1 + 2 as hypercoagulation biomarkers. DP and mortality were monitored during follow-up. Results: A prospective cohort of 462 colorectal and 164 gastric cancer patients was available for analysis. After 6 months, DP occurred in 148 patients, providing a cumulative incidence of 24.8% (21.4–28.4). D-dimer and TG endogenous thrombin potential (ETP) were identified as independent risk factors for 6m-DP by multivariate Fine–Gray proportional hazard regression model corrected for age, cancer site, and >1 metastatic site. After 1 year, we observed an OS of 75.7% (71.9–79.0). Multivariate Cox regression analysis corrected for age, site of cancer, and performance status identified D-dimer and ETP as independent risk factors for 1y OS. Patients with one or both hemostatic parameters above the dichotomizing threshold were at higher risk for both 6m-DP and 1-year mortality. Conclusion.: in newly diagnosed metastatic GI cancer patients, pretreatment ETP and D-dimer appear promising candidate biomarkers for predicting 6m-DP and 1y OS. In this setting, for the first time, the role of TG as a prognostic biomarker emerges in a large prospective cohort.

## 1. Introduction

Gastrointestinal (GI) cancers represent 26% of the global cancer incidence and more than one third of all cancer-related deaths and they are characterized by high invasive and metastatic potential [1]. The clinical benefits of all innovative and wide-ranging treatments developed over the years differ critically among individuals and, in many cases, patient’s prognosis remains poor [2]. For all these reasons, there is a great need for new prognostic biomarkers for prediction of survival and predictive biomarkers for treatment efficacy or toxicity.

It is well known that there is a close and mutual relationship between cancer and blood coagulation.

Cancer favors blood coagulation activation with a consequent appearance of a systemic and subclinical hypercoagulable state that can be detected as alterations in laboratory coagulation tests [3,4]. Cancer-associated hypercoagulability increases the risk of thromboembolic complications, which do occur at a high rate in cancer compared to the non-cancer population [5,6]. On the other hand, hypercoagulability influences tumor biology, favoring tumor growth and metastatic dissemination in a complex and multifactorial way [7].

Based on this reciprocity between cancer and coagulation, in the last decades, many studies have been performed with the aim of evaluating the prognostic role of hemostatic biomarkers in cancer disease [8]. The majority of available publications are focused on specific cancer types and report a significant relationship between hemostatic biomarkers and overall survival (OS), disease-free survival (DFS), and progression-free survival (PFS). Particularly, in colorectal cancer (CRC) patients undergoing surgery or receiving chemotherapy, pretreatment fibrinogen and D-dimer levels significantly predicted for response to therapy and poor survival [9,10,11,12]. Similarly, in operable gastric cancer (GC), high preoperative levels of D-dimer and fibrinogen were associated with peritoneal dissemination and poor survival [13,14,15], while, in metastatic GC, D-dimer levels predicted for response to chemotherapy and OS [16].

Regrettably, the majority of these studies were single-center and retrospective, often involving small groups of patients, and, most importantly, not specifically designed to address the impact of thrombotic biomarkers on cancer outcomes. Moreover, they were mainly conducted on patients with localized tumors awaiting cytoreductive surgery and adjuvant therapy, while little has been investigated in patients with a new diagnosis of advanced metastatic cancer.

Among biomarkers of hypercoagulability, thrombin generation (TG) assay is unique, being a global hemostatic test sensitive to several genetic and acquired thrombophilic states, including solid and hematological malignancies [17,18,19]. TG parameters, together with other coagulation biomarkers, predicted a breast cancer diagnosis [20]. Regarding disease prognosis, the TG potential has been included in a risk-assessment model for prediction of an early disease recurrence in operated breast cancer patients undergoing adjuvant chemotherapy [21]. Up to now, no study has been published on the prognostic role of TG in gastrointestinal cancer.

Therefore, despite all the encouraging results reported so far, new data coming from large prospective cohorts are needed to strenghthen the role of hemostatic biomarkers in cancer prognosis.

In this respect, we analyzed a large prospective cohort of patients with newly diagnosed metastatic GI cancer enrolled in the ongoing multicenter observational HYPERCAN study [22] (ClinicalTrials.gov ID#NCT02622815), to evaluate whether prechemotherapy values of TG potential and hypercoagulation biomarkers may predict for 6-months disease progression (6m-DP) and for 1-year overall survival (1y OS). The HYPERCAN study represents the first large prospective study specifically designed to demonstrate association between biomarkers of hypercoagulability and prognosis in cancer patients.

## 2. Materials and Methods

### 2.1. Study Design and Population

The study population included 626 newly diagnosed metastatic GI patients enrolled between May 2012 and November 2019 in the HYPERCAN study. Patients were recruited at 8 Italian oncology units (Appendix A). Inclusion criteria were having newly diagnosed metastatic CRC or GC (stage TXNXM1) and being a candidate to systemic chemotherapy. Exclusion criteria were: acute medical illnesses, hospitalization, therapeutic anticoagulation, and life expectancy < 3 months. Age, gender, body mass index (BMI), performance status, relevant comorbidities, prophylactic use of anticoagulants (any reason different to cancer), tumor type and size, histological subtype, lymph node status, tumor grading (i.e., Nottingham score), and tumor biological characteristics were recorded at the enrollment. Patients were then followed up for at least 5 years and clinical information on any antitumor treatment and clinical response were recorded. The median follow-up time of the cohort was 472 days (57–1615 days).

### 2.2. Ethical Statement

The study protocol has been approved by the local Ethics Committee (Comitato Etico della Provincia di Bergamo, del. 146, 1 February 2012). All subjects provided informed written consent. Consent was also obtained for data recording, collection, and storage of blood samples to allow regulatory monitoring, statistical analysis, and publication of results. The ethical conduct of the study is regulated by the last revision of the Helsinki Declaration. The study was coordinated in the Department of Immunohematology and Transfusion Medicine, Hospital Papa Giovanni XXIII Bergamo, Italy.

### 2.3. Collection of Blood Samples and Plasma Preparation

Fasting peripheral venous blood samples were collected into 6 mL vacutainer tubes containing 0.109 M Na_3_ citrate (9:1 *v*/*v*; Becton Dickinson, Vacutainer, Plymouth, UK) [23]. Platelet-poor plasma was obtained by double centrifugation at 2600× *g* for 15 min at 25 °C and stored at −80 °C [21,24]. Nameless samples were centrally tested at the Laboratory of Hemostasis and Thrombosis (Hospital Papa Giovanni XXIII, Bergamo, Italy). Blood sample collection, processing, and storage were performed in accordance to standardized procedure and international recommendations [25,26].

### 2.4. Thrombin Generation Potential and Plasma Hemostatic Biomarkers

TG was evaluated by the calibrated automated thrombogram method at 5pM Tissue Factor (CAT assay, Stago) [17,27]. All plasma samples were tested in duplicate. The following parameters of TG curve were considered: lag-time, endogenous thrombin potential (ETP), peak height (peak), and time to peak (ttp). Plasma levels of D-dimer (HemosIL D-dimerHS, Werfen Group) and fibrinogen (QFA thrombin, Werfen Group) were measured on an automated coagulometer analyzer, according to manufacturer procedure (ACL TOP500, Werfen Group). Prothrombin fragment 1 + 2 (F1 + 2) was measured using commercially available ELISA (Siemens).

### 2.5. Study Outcomes

Study outcomes were the occurrence of a documented DP within 6 months from the starting of chemotherapy treatment and 1y OS.

### 2.6. Statistical Analysis

In the descriptive statistics, categorical data were summarized as frequencies and proportion, while continuous variables were categorized as mean and standard deviation or median and 5th–95th percentile range, depending on their distribution. Differences in proportion of categorical variables were tested by Chi^2^ test. Differences between groups were tested by Student t-test for normally distributed variables and by Mann–Whitney test for variables not normally distributed. Laboratory variables were dichotomized according to a cut-off value, determined with receiver operating characteristic (ROC) curves, and chosen to optimize both outcomes (6m-DP and 1y OS). Multivariate proportional hazard regression analyses (Fine–Gray with death as competing risk for 6m-DP and Cox for 1y OS) were performed with a backward variable selection, including clinical (age, gender, BMI, smoke, comorbidities, use of anticoagulant/antiplatelet agents, Eastern Cooperative Oncology Group Performance Status (ECOG-PS), and number of metastases) and laboratory covariates (F1 + 2, D-dimer, fibrinogen, and TG parameters). Assuming the beginning of chemotherapy as baseline time, crude cumulative incidences of 6m-DP were estimated using death as competing risk and compared by Gray test, while survival functions of OS were estimated using the Kaplan–Meier method and compared by log-rank test. Missing values have been handled with omission (samples with invalid data are discarded from analysis). Statistical analysis has been performed using R v4.1 software.

## 3. Results

### 3.1. Characteristics of Study Population

The study population included 462 CRC and 164 GC with a median age of 66 years (range: 26–87 years), 61% of which were male (Table 1). About 85% of subjects had an ECOG-PS ≤ 1, 68.5% had comorbidities, and 69.7% had one or more cardiovascular risk factors, including diabetes, hypertension, hypercholesterolemia, and atherosclerosis. Use of antiplatelet (*n* = 77) or prophylactic anticoagulant (*n* = 32) treatment at enrollment was not different between GC and CRC patients. Seventeen patients had a history of thrombosis, consisting in eight venous (four GC and four CRC) and nine arterial (four GC and five CRC) thrombosis. A total of 303 patients had more than one metastatic site, particularly GC patients (*p* < 0.001).

### 3.2. Hematological and Hemostatic Parameters

Most patients presented with hematological parameters in the normal range, except for hemoglobin (<11.5 g/dL in 35.2%) (Table 2). In addition, GC patients had statistically significant (*p* < 0.01) lower RBC count and hemoglobin values compared to CRC patients. Regarding hemostatic parameter analysis, a large proportion of patients presented F1 + 2 (24.6%), D-dimer (63.8%), and fibrinogen (52.8%) values above the normal range, with GC patients showing the highest D-dimer levels. Similarly, TG potential was increased, with peak above the normal range in 28.6% and ETP in 15.6% of patients (Table 2).

### 3.3. 6-Month DP and Association with Hematological and Hemostatic Parameters

After the beginning of chemotherapy, 6m-DP occurred in 148 patients (Table 1), with a cumulative crude incidence of 24.8% (CI 95% [21.4–28.4]). Patients who experienced a DP showed, at enrollment, increased WBC count (*p* = 0.036) and D-dimer level (*p* < 0.001) compared to no-DP patients (Table 3).

Multivariate regression analysis was performed for 6m-DP (with death as competing risk) starting from a full model including all laboratory and clinical-pathological covariates reported in the material and methods section. After backward elimination, the multivariate model, stratified for age, site of tumor, and number of metastases, identified D-dimer > 420 ng/mL (SHR = 1.4, CI 95% [1.1–2.1]; *p* = 0.047) and TG ETP > 1700 nM∙min (SHR = 1.6, CI 95% [1.1–2.2]; *p* = 0.014) as independent risk factors for 6m-DP (Table 4).

### 3.4. One-Year OS and Association with Hematological and Hemostatic Parameters

After 1 year, 139 deaths were recorded, providing an OS cumulative incidence of 75.7% (71.9–79.0). Patients who died in the first year were characterized by significantly lower RBC and hemoglobin levels (*p* < 0.001) and higher WBC count (*p* < 0.001), F1 + 2 (*p* = 0.004), D-dimer (*p* < 0.001), and fibrinogen (*p* = 0.023) compared to patients who survived, together with higher TG ETP and peak (*p* < 0.01) (Table 3).

In a model stratified for age, site of cancer, and ECOG-PS, we found that D-dimer > 420 ng/mL (HR = 2.4, CI 95% [1.5–3.7]; *p* < 0.001) and ETP > 1700 nM∙min (HR = 2.0, CI 95% [1.3–3.1]; *p* = 0.002) were independently associated with risk of death during the first year (Table 4).

### 3.5. Patient Stratification for 6m-DP and 1y OS Risk According to D-Dimer and TG Parameters

**Figure 1:**
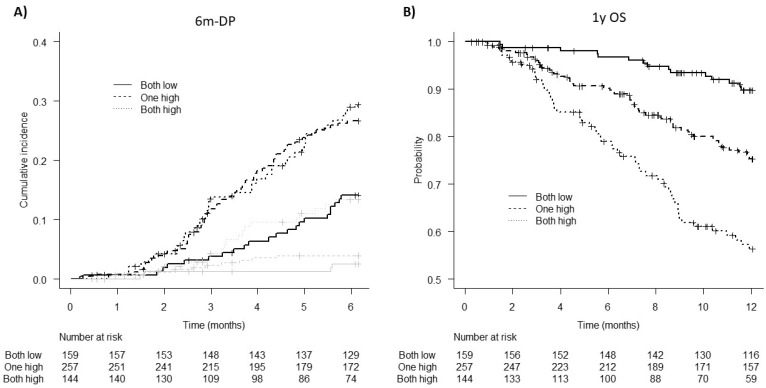
(**A**) Crude cumulative incidence of 6m-DP and (**B**) Kaplan–Meier estimates of 1-year OS according to risk groups based on D-dimer and TG ETP levels. Continuous line = low risk (both under threshold), dashed line = intermediate risk (at least one over threshold), and dotted line = high risk (both over threshold). Grey lines are corresponding estimates of the competing event. 6m-DP: 6-month disease progression, 1y OS: 1-year overall survival, TG: thrombin generation, ETP: endogenous thrombin potential.

Figure 1 reports crude cumulative incidences of 6m-DP and Kaplan–Meier estimates of 1y OS according to patient stratification in three risk categories based on D-dimer and TG ETP: low risk (both D-dimer and TG ETP low), intermediate risk (D-dimer or ETP high), and high risk (both D-dimer and ETP high). As shown in Figure 1A, having at least one or both parameters above the threshold identified two groups of patients at equivalent risk of 6m-DP (27% intermediate, 29.8% high) but each at significantly (*p* < 0.01) higher risk as compared to the low-risk group (14.1%) (SHR = 2.1 [1.3–3.4] low vs. intermediate; SHR = 2.3 [1.4–3.9] low vs. high). Furthermore, this classification appears to be very efficient in predicting the risk of 1y OS, as it discriminates well patients at low risk of death (OS = 89.8%), at intermediate risk (OS = 75.2%; HR = 2.7 [1.5–4.8], *p* = 0.001 vs. low), and at high risk (OS = 56.3%; HR = 5.5 [3.1–9.8] *p* < 0.001 vs. low) after 1 year from diagnosis (Figure 1B).

## 4. Discussion

In this prospective cohort of 626 metastatic GI cancer patients, we investigated on the capability of plasma hemostatic biomarkers, measured before starting chemotherapy, to predict for early DP (i.e., 6 months) and 1-year OS. In this cohort, 6m-DP was observed in 25.6% of patients with metastatic GI, mostly in GC (46.2%) compared to CRC (18.8%), and consistently with the literature where the gastric tumor site is at high risk for both DP and mortality [28].

Our analysis showed that elevated D-dimer and TG ETP values in a model corrected for tumor site and the presence of more than one metastatic site were independent risk factors for 6m-DP. Results on D-dimer are consistent with those from other studies, which reported an association between elevated pretreatment plasma D-dimer levels and a worse prognosis in patients with different types of carcinomas [29,30,31], including pancreatic, esophageal, gastric, and colorectal cancer [16,32,33,34,35], and with a recent meta-analysis involving 5928 GI patients showing that pretreatment D-dimer levels predicted for poor prognosis [36]. Less is known about the role of TG in this setting and our study appears to be the first in the literature to demonstrate the prognostic role of TG for DP in metastatic GI. Indeed, today, TG was found useful only for prediction of early recurrence (within 2 years) in resected breast cancer patients at high risk of relapse who started anticancer chemotherapy [21,37].

In our cohort, fibrinogen failed to be identified as an independent risk factor for 6m-DP. Some studies [10,38] had investigated the prognostic role of fibrinogen in cancer patients but for different outcomes (i.e., DFS) and in the different setting of resected patients, while there is little or no evidence in patients with metastatic GI cancer.

Overall survival evaluated at 1 year after enrollment showed a total of 139 deaths, providing a 75.2% cumulative incidence, with a better prognosis in CRC patients compared to GC (83.0% vs. 51.1%). As for 6m-DP, elevated values (over cutoffs) of D-dimer and TG ETP were independent risk factors for 1y OS. In the literature, the pretreatment D-dimer levels have already been associated with poor survival in resected GI patients [14,39] and in advanced or relapsing CRC patients [40] but not specifically in metastatic cancer. Similarly, no data are available so far on the prognostic role of TG, in particular of the ETP parameter, as an independent risk factor for mortality in patients with metastatic GI cancer. In our analysis, fibrinogen appeared not to be promising for predicting 1y OS. Again, data available in the literature mainly referred to resected patients in which, according to recent meta-analysis on 11 studies on GC patients, preoperative fibrinogen predicted for OS and recurrence-free survival [41].

A possible limitation of this study lies in the fact that data on type of chemotherapy, molecular markers (HER-2), and genetic mutations (KRAS, NRAS, and BRAF) were not available for all patients and, therefore, not included in the analysis. In the future, it will be very important to evaluate the contribution of these factors in relation to patient prognosis and to use them for predicting therapy response and survival. Another limitation may consist in that, in this analysis, biomarker measurements are single-point and not repeated during follow-up. This does not constitute a real limitation for us, because our aim is to provide clinicians with tools to identify high-risk patients specifically before the start of chemotherapy. Finally, it is often reported that CAT assay, being a semiautomatic system, may be affected by operator variability. Indeed, our group has recently validated TG results from CAT assay with a fully automated system in breast cancer patients, obtaining excellent outcomes [37].

## 5. Conclusions

In conclusion, two biomarkers of hemostasis, D-dimer and TG ETP, allowed the identification of patients at high risk for both 6m-DP and death. Evaluating the prognosis for a patient with cancer is of the greatest clinical interest and can provide useful information on the type and intensity of the anticancer treatment to be administered. In the future, these results need to be validated in an independent cohort of patients, and then could be used for the creation of a score for identification right from the tumor diagnosis of patients into risk categories based on the outcomes of the disease, with the ultimate aim of personalizing the anticancer treatment.

## Figures and Tables

**Table 1:** Characteristics of all GI patients and according to tumor site (GC vs. CRC).

	Total (*n* = 626)	GC (*n* = 164)	CRC (*n* = 462)	*p* Value
Gender (*n*)	383 M/243 F	105 M/59 F	278 M/184 F	0.385
Age (years, median, min–max)	66 (26–87)	65 (26–84)	67 (29–87)	0.248
ECOG-PS (*n*, %)				
0	338 (54)	77 (47)	261 (56.5)	**0.015**
1	191 (30.5)	61 (37.2)	130 (28.1)	
2	35 (5.6)	14 (8.5)	21 (4.5)	
NA	62 (9.9)	12 (7.3)	50 (10.8)	
Comorbidities (*n*, %)				
Yes	429 (68.5)	115 (70.1)	314 (68.0)	0.737
No	192 (30.7)	49 (29.9)	143 (31.0)	
NA	5 (0.8)	-	5 (1.1)	
Cardiovascular risk * (*n*, %)				
Yes	299 (69.7)	73 (63.5)	226 (72.0)	0.073
No	128 (29.8)	42 (36.5)	86 (27.4)	
NA	2 (0.5)	-	2 (0.6)	
Antiplatelet therapy (*n*, %)				
Yes	77 (12.3)	19 (11.6)	58 (12.6)	0.706
No	543 (86.7)	145 (88.4)	398 (86.1)	
NA	6 (1.0)	-	6 (1.3)	
Anticoagulant therapy (*n*, %)				
Yes	32 (5.1)	9 (5.5)	23 (5.0)	0.826
No	588 (93.9)	155 (94.5)	433 (93.7)	
NA	6 (1.0)	-	6 (1.3)	
History of venous thrombosis (*n*, %)				
Yes	8 (1.3)	4 (2.4)	4 (0.9)	0.122
No	520 (83.1)	134 (81.7)	386 (83.5)	
NA	98 (15.7)	26 (15.9)	72 (15.6)	
History of arterial thrombosis (*n*, %)				
Yes	9 (1.4)	4 (2.4)	5 (1.1)	0.207
No	519 (82.9)	134 (81.7)	385 (83.3)	
NA	98 (15.7)	26 (15.9)	72 (15.6)	
Number of metastatic sites (*n*, %)				
1	305 (48.7)	57 (34.8)	248 (53.7)	**<0.001**
>1	303 (48.4)	103 (62.8)	200 (43.3)	
NA	18 (2.9)	4 (2.4)	14 (3.0)	
6m-DP (*n*, %)	148 (23.6)	67 (40.9)	81 (17.5)	**<0.001**
Time to 6m-DP (days, median, 5th–95th)	104 (40–175)	90 (36–178)	112 (46–171)	0.341
1y-death (*n*, %)	139 (22.2)	67 (40.9)	72 (15.6)	**<0.001**
Time to 1y-death (days, median, 5th–95th)	191 (45–351)	195 (47–321)	188 (33–355)	0.958

Data are presented as number (percentage) or median (5th–95th). For categorical variables, *p* is the statistical significance from chi-square test for equality of distribution between groups. For continuous variables, P is the statistical significance by Mann–Whitney test. GI: gastrointestinal, GC: gastric cancer, CRC: colorectal cancer, ECOG-PS: Eastern Cooperative Oncology Group Performance Status, 6m-DP: 6 months disease progression, NA: not available. * One or more among diabetes, hypertension, hypercholesterolemia, and atherosclerosis.

**Table 2:** Hematological parameters and plasma levels of coagulation biomarkers according to site of tumor.

	Total (*n* = 626)	GC (*n*= 164)	CRC (*n* = 462)	*p* Value	Ref
WBC (10^9^/L)	7.1 (4.2–13.0)	6.9 (3.9–12.6)	7.2 (4.3–13.1)	0.387	4.5–11
RBC (10^12^/L)	4.48 (3.54–5.35)	4.33 (3.24–5.08)	4.52 (3.61–5.40)	**0.010**	3.9–5.0
Hemoglobin (g/dl)	12.3 (9.4–15.3)	12.0 (9.1–15.0)	12.3 (9.4–15.4)	**0.007**	11.5–14.4
Platelets (10^9^/L)	263 (134–522)	252 (115–507)	265 (140–523)	0.149	150–450
F 1 + 2 (pmol/L)	343 (161–862)	361 (151–1337)	337 (163–745)	0.181	215 (126–478)
D-dimer (ng/mL)	375 (104–2108)	462 (121–3114)	350 (99–1872)	**0.003**	108 (44–283)
Fibrinogen (mg/dl)	410 (235–725)	400 (207–726)	410 (247–704)	0.426	150–400
TG lag time (min)	3.1 (2.0–5.5)	3.2 (2.0–5.8)	3.1 (2.0–5.5)	0.543	3.1 (2.2–4.45)
TG ETP (nM∙min )	1702 (962–2601)	1689 (951–2602)	1706 (963–2600)	0.413	1516 (970–2168)
TG peak (nM)	344 (117–524)	360 (122–554)	341 (115–521)	0.253	237 (128–404)
TG ttPeak (min)	5.7 (4.0–9.8))	5.7 (3.8–10.1)	5.7 (4.0–9.8)	0.498	6.7 (4.7–8.8)

Data are presented as median (5th–95th). Normal reference values for D-dimer, F1 + 2, and TG are internally derived. P is the statistical significance by Mann–Whitney test. GC: gastric cancer; CRC: colorectal cancer; WBC: white blood cells, RBC: red blood cells, F1 + 2: prothrombin fragment 1 + 2, TG: thrombin generation. ETP: endogenous thrombin potential. ttPeak: time to peak.

**Table 3:** Hematological parameters and plasma levels of coagulation biomarkers according to 6m-DP and 1-year OS.

	6m-DP	No 6m-DP	1y Death	1y OS
WBC (10^9^/L)	7.6	7.0 *	8.5	6.8 ***
	(4.3–12.6)	(4.2–13.1)	(4.2–15.6)	(4.2–12.5)
RBC (10^12^/L)	4.41	4.49	4.30	4.53 **
	(3.47–5.39)	(3.57–5.34)	(3.20–5.27)	(3.60–5.42)
Hemoglobin (g/dL)	12.2	12.3	11.6	12.4 **
	(9.4–15.7)	(9.2–15.1)	(9.4–15.6)	(9.4–15.2)
Platelets (10^9^/L)	270	262	294	259
	(148–597)	(133–508)	(133–507)	(138–526)
F1 + 2 (pmol/L)	358	337	382	337 **
	(166–1164)	(160–806)	(172–1298)	(160–813)
D-dimer (ng/mL)	460	360 **	584	342 ***
	(141–2686)	(94–1868)	(154–3694)	(95–1578)
Fibrinogen (mg/dL)	418	408	443	402 *
	(241–726)	(234–704)	(225–742)	(239–704)
TG lag time (min)	3.2	3.1	3.4	3.1 ***
	(2.1–5.7)	(2.0–5.4)	(2.1–6.1)	(2.0–5.3)
TG ETP (nM∙min)	1783	1687	1885	1673 **
	(843–2595)	(962–2605)	(1052–3122)	(955–2535)
TG peak (nM)	362	339	378	337 **
	(104–527)	(115–525)	(127–590)	(112–502)
TG ttPeak (min)	5.9	5.7	5.9	5.7
	(4.2–10.2)	(4.0–9.7)	(4.3–9.8)	(4.0–9.8)

Data are presented as median (5th–95th). P is the statistical significance by Mann–Whitney test (* *p* < 0.05, ** *p* < 0.01, *** *p* < 0.001). WBC: white blood cells, RBC: red blood cells, F1 + 2: prothrombin fragment 1 + 2, TG: thrombin generation, ETP: endogenous thrombin potential, 6m-DP: 6 months disease progression, 1y OS: 1-year overall survival.

**Table 4:** Proportional hazard regression analysis for 6m-DR and death during first year from diagnosis.

	6m-DP			1y OS		
	SHR	CI	*p* Value	HR	CI	*p* Value
D-dimer > 420 ng/mL	1.44	1.01–2.06	0.047	2.41	1.53–3.80	<0.001
TG ETP > 1700 nM∙min	1.57	1.09–2.25	0.014	2.02	1.30–3.14	0.002

Observation = 560. Model for 6m-DP by Fine–Gray proportional hazard regression is stratified for age, site of tumor, and number of metastases. Model for 1y OS by Cox proportional hazard regression is stratified for age, site of tumor, and ECOG-PS. 6m-DP = 6-month disease progression. 1y OS = 1-year overall survival. SHR: sub-distribution hazard ratio. HR: hazard ratio. CI: confidence interval. TG: thrombin generation. ETP: endogenous thrombin potential.

## Data Availability

The data presented in this study are available on request from the corresponding author.

## Appendix A

The members of the HYPERCAN Study (by centers, all in Italy) are the following: Coordinating Center: Immunohematology and Transfusion Medicine, Hospital Papa Giovanni XXIII, Bergamo: Falanga Anna, Bolognini Silvia, Gamba Sara, Giaccherini Cinzia, Marchetti Marina, Russo Laura, Schieppati Francesca, Tartari Carmen Julia, Ticozzi Chiara, Verzeroli Cristina. Participants: *IRCCS Humanitas Institute, Rozzano*: Santoro Armando, Masci Giovanna. *IRCCS National Cancer Institute, Milan*: De Braud Filippo, Celio Luigi, Martinetti Antonia, Nichetti Federico. *Hospital Papa Giovanni XXIII, Bergamo, Oncology Unit*: Tondini Carlo. Medical oncologist: Labianca Roberto. *Hospital San Filippo Neri, Rome*: Gasparini Giampietro, Sarmiento Roberta, Gennaro Elisabetta. *Hospital San Giovanni, Rome*: Minelli Mauro. *Hospital Treviglio-Caravaggio, Treviglio*: Barni Sandro, Petrelli Fausto, Ghilardi Mara. *Policlinico San Marco, Gruppo San Donato, Zingonia-Bergamo*: D’Alessio Andrea, Cecchini Sara. IRCCS *Cancer Institute Giovanni Paolo II, Bari*: Giuliani Francesco.
